# Tackling injustices of occupational lung disease acquired in South African mines: recent developments and ongoing challenges

**DOI:** 10.1186/s12992-018-0376-3

**Published:** 2018-06-28

**Authors:** Barry Kistnasamy, Annalee Yassi, Jessica Yu, Samuel J. Spiegel, Andre Fourie, Stephen Barker, Jerry M. Spiegel

**Affiliations:** 1 Department of Health, Johannesburg, South Africa; 2 School of Population and Public Health (SPPH), University of British Columbia (UBC), 430-2206 East Mall, Vancouver, BC V6T 1Z3 Canada; 3 Centre of African Studies, University of Edinburgh, Edinburgh, UK

**Keywords:** Mining, South Africa, Workers’ compensation, Tuberculosis, Occupational lung disease, Social justice, Social protection, Southern Africa, Migrant workers, Underfunding

## Abstract

**Background:**

South Africa’s mineral resources have produced, and continue to produce, enormous economic wealth; yet decades of colonialism, apartheid, capital flight, and challenges in the neoliberal post-apartheid era have resulted in high rates of occupational lung disease and low rates of compensation for ex-miners and their families. Given growing advocacy and activism of current and former mine workers, initiatives were launched by the South African government in 2012 to begin to address the legacy of injustice. This study aimed to assess developments over the last 5 years in providing compensation, quantify shortfalls and explore underlying challenges.

**Methods:**

Using the database with compensable disease claims from over 200,000 miners, the medical assessment database of 400,000 health records and the employment database with 1.6 million miners, we calculated rates of claims, unpaid claims and shortfall in claim filing for each of the southern African countries with at least 25,000 miners who worked in South African mines, by disease type and gender. We also conducted interviews in Johannesburg, Eastern Cape, Lesotho and a local service unit near a mine site, supplemented by document review and auto-reflection, adopting the lens of a critical rights-based approach.

**Results:**

By the end of 2017, 111,166 miners had received compensation (of which 55,864 were for permanent lung impairment, and another 52,473 for tuberculosis), however 107,714 compensable claims remained unpaid. Many (28.4%) compensable claims are from Mozambique, Lesotho, Swaziland, Botswana and elsewhere in southern Africa, a large proportion of which have been longstanding. A myriad of diverse systemic barriers persist, especially for workers and their families outside South Africa. Calculating predicted burden of occupational lung disease compared to compensable claims paid suggests a major shortfall in filing claims in addition to the large burden of still unpaid claims.

**Conclusion:**

Despite progress made, our analysis reveals ongoing complex barriers and illustrates that the considerable underfunding of the systems required for sustained prevention and social protection (including compensation) needs urgent attention. With class action suits in the process of settlement, the globalized mining sector is now beginning to be held accountable. A critical rights-based approach underlines the importance of ongoing concerted action by all.

## Background

The extensive mineral resources of South Africa have produced, and continue to produce, enormous wealth in aggregate economic terms; yet decades of colonialism, apartheid, flight of capital from the region, and challenges in the neoliberal post-apartheid era have left hundreds of thousands of mine workers in southern Africa with occupational lung disease, along with associated health, economic and social consequences [1–10]. In an article in *Globalization and Health* almost 10 years ago analyzing tuberculosis in miners who had worked in South African mines, Basu and colleagues called for immediate actions to address the health and social inequities created by the mining sector in South Africa, noting that “the mining industry is not paying the full price” of the lung disease it was creating; they also drew attention to “a critical absence of a focal point of government leadership” [11].

The issue of redressing injustices linked to unhealthy and unfair mining conditions has risen to the top of the political agenda in South Africa in recent years. This was again illustrated in the words of the new President of South Africa, Cyril Ramaphosa, speaking in February 2018 about the critical importance of “healing and atonement” following the 2012 massacre of 34 striking mineworkers [12]. Silicosis, tuberculosis and other occupational lung diseases associated with mining have long existed as unaddressed threats to South African and migrant populations across southern Africa who came to work in mines in search of a decent life; rectifying such injustices requires attending to both the experiences of a diverse range of individuals as well as structural factors and processes that can create barriers to meaningful redress.

Moving beyond scholarship on the impact of tuberculosis and other lung diseases associated with mining in the region, there is a critical need to grapple with what it means to create a more equitable compensation system for those from across southern Africa who worked in South African mines – for corporations that are primarily global in their ownership and trading relations. Our study discusses some recent initiatives to tackle injustices for ex-miners, assessing accomplishments, documenting shortfalls and analyzing persisting challenges. We draw on first-hand knowledge, key informant interviews as well as never-previously published data from a database of over 300,000 miners and ex-miners who filed compensation claims, linking these to recently published employment data [13] on miners from across southern Africa. Below we first briefly present some essential historical background, then the methods and results of our analysis of recent developments.

### Colonial labour practices and grassroots struggle

From the late 1800s, when colonial British interests discovered the mineral riches of South Africa, a pattern of employment practices was set in motion that would lead to health consequences beyond those inherent in mining worldwide [14]. Initial reliance on skilled workers triggered a migration of experienced deep shaft miners from the United Kingdom, many of whom brought with them tuberculosis acquired during Britain’s Industrial Revolution [15]. The labour-intensive methods pursued in South Africa gold mining triggered the additional colonial practice of importing workers from neighbouring areas, a pattern that has diminished but still persists [13]. With the heavy toll generated by exposure to silica dust, along with difficult working and living conditions, hundreds of thousands who worked in the mines of South Africa not only contracted chronic dust-induced diseases but also incurred high rates of tuberculosis [16]. As explained by Murray and colleagues [9], silica exposure is associated with an increased lifelong risk of tuberculosis even in the absence of silicosis, while increased HIV transmission stimulated by migration and single-sex living compounds has further driven the incidence of tuberculosis. Indeed those who worked in South African gold mines have incurred rates of tuberculosis up to ten times greater than the general population [11]. In the 1970s, most of these individuals (an estimated 55%) had left families in Botswana, Lesotho, Mozambique, Swaziland and elsewhere in southern Africa to work in these mines [13]. Coercive policies and legislation restricting access to land and means of production in these countries were among measures forcing male labourers to migrate to become mine workers, often disrupting family ties and undermining the rural black agricultural economy in order to maintain sources of cheap labour [17]. Health concerns across the region were then compounded when these migrant miners returned home ill, putting other household or community members at risk of transmission of undetected or inadequately treated tuberculosis, particularly with drug-resistant forms of the disease [11].

Meanwhile, South Africa’s Occupational Diseases and Mines and Works Act of 1973 mandated compensation of miners who contracted occupational lung disease, including silicosis, tuberculosis, and chronic obstructive pulmonary disease, entitling ex-miners to biennial benefit medical examinations (BMEs). Although this compensation legislation was considered progressive at the time, it applied essentially only to white miners; circumstances for black workers - who constituted the overwhelming majority of exposed workers – were quite different, with the extent of their disease obscured by limited diagnosis and treatment services in their home regions providing implicit rationale for failure to take action [18]. With the anti-apartheid struggle succeeding through intense grassroots efforts and international support to bring democracy to South Africa in 1994, the overtly racial clauses governing compensation were removed. However, challenges in the post-apartheid era continued to leave hundreds of thousands of ex-miners with occupational lung disease, no access to health assessments and no financial compensation [9, 10, 19], with barriers especially problematic for migrant workers [20].

Research documenting occupational lung disease attributable to South African mines accompanied growing advocacy from ex-miners’ associations for “social protection” benefits including compensation for occupational disease. In 1997, Dr. Ahmed Randeree,^1^ a long-time anti-apartheid activist forced to live abroad, returned to South Africa to assist the then Deputy Director General of Health in the Northern Cape Province, to begin the first large assessment of former asbestos workers in Prieska. This screening project contributed to promoting litigation to establish the Asbestos Relief Trust in 2003 and the Kgalagadi Relief Trust in 2006. After the historical case by asbestosis and mesothelioma sufferers, Richard Spoor [21] predicted that “a wave of litigation against the industry should be anticipated...as former miners and their advocates turn to the courts for compensation.” Indeed, within a few years, ex-miners associations had intensified their efforts; in 2009, this led to a class action suit focusing on silicosis [22]; subsequently, another class action was launched covering silicosis and tuberculosis [23]. In March 2016, after a long legal battle, a landmark settlement was reached between two of the major mining companies and 4365 former mine workers – establishing a trust valued at approximately 400 million Rand for silicosis sufferers and paving the way for further court action [24].

### Responding to injustice

Historically, the governance of compensation for ex-miners consisted of a certification committee based at the Medical Bureau of Occupational Disease (MBOD) that focused on adjudicating as to whether the technical requirements of diagnosis and exposure were met. There was no overall accountability for remediating the burden of uncompensated ex-miners, as if those affected across southern Africa had full knowledge of their rights and access to resources to pursue these despite the extreme power imbalances inherent in colonial relationships. The revenue structure for compensation has been (and remains) problematic as the levies on the mining companies which generated the diseases only cover worker income protection and not the administration of the system, provision of BMEs or healthcare, thereby externalizing such cost to individuals, families and government – including other countries in southern Africa. Although correction to this chronic underfunding had been strongly resisted by mining companies, notably in the 2010 opposition of the Chamber of Mines to increasing levies on the industry to enable this [8, 10], the emergence of class actions and activism introduced new urgencies to address this issue.

London and Schneider [25] argued that proponents of neoliberal globalization often promote rhetoric of requiring nations to fulfil human rights obligations while paradoxically disempowering the ability of states to deliver health and social protection. Noting that the World Conference on Human Rights [26] acknowledges that socioeconomic rights are inextricably linked to civil and political freedoms, they proposed a rights lens that encourages civil society action to hold public officials accountable. With the courts deliberating and labour activism intensifying, the South African government initiated new processes in 2012, notably the appointment of a Compensation Commissioner for Occupational Diseases (CCOD) with a mandate to address the ongoing inadequacies. Mining companies, the World Bank, UK Aid and the Global Fund subsequently were brought together with government and others to begin to discuss ways to raise the revenue needed for a sustainable new service delivery model, with commitments secured for at least this transition stage. The very establishment of an explicit mandate – a focal point - to drive improvements in system management and service delivery to address the longstanding injustices marked a fundamental change in purpose and accountability – and created a basis for considering the efficacy of steps to achieve this.

Adopting a critical right-based lens, this article responds to calls for analysis of efforts to address past inequities in southern Africa [27] by assessing what has been accomplished in the past 5 years to provide health services and social protection for miners who developed occupational lung disease. Situated in the context of a globalized mining industry, this article documents and quantifies progress made, while analyzing shortfalls and calling attention to the next steps urgently needed to address underlying injustices.

## Methods

We draw from participant observation and auto-reflection of the first author (BK), who himself has been serving as the CCOD over the 2012–2017 period of this study, as well as the experience of co-author AV, who is in charge of developing and managing the CCOD database. Review of numerous documents from government, non-governmental organizations, the media and the Chamber of Mines supplemented the analysis by our interdisciplinary international team which included a critical African Studies scholar with expertise in the mining sector (SJS), a political economist with particular interest in globalization (JS), an occupational health researcher with a background in compensation for ex-miners (AY) and a statistician (SB).

The auto-reflections and document review were then triangulated with information from twelve interviews conducted by a doctoral student from a Canadian university (JY) – decreasing the likelihood that interviewees would be reticent to express their concerns. These interviews, conducted in July 2016 following an initial planning trip in which AY and JY prepared this work, were held in Johannesburg as well as in the Eastern Cape and in Lesotho, in addition to at a local decentralized service unit, the Carletonville “One Stop” Service centre, located near one of the mine sites. The interviews ranged from 30 to 45 min with interviewees chosen by purposive sampling of known key informants, extended through snowball sampling. Interviewees included staff from the CCOD and the MBOD as well as unions and mining company representatives, and comprised personnel with responsibility for health and safety as well as payroll, finances, outreach and revitalization, among others. The semi-structured interview guide included pre-determined themes but allowed for new themes to emerge. All interviews were conducted in English, audio recorded, and supplemented with handwritten field notes. Inductive content analysis was used, which included open coding, with coding done iteratively throughout the data collection and analysis processes, based on connections with the research questions and existing literature.

The centre-piece of our analysis derives from the database established by CCOD/MBOD as described below, with newly compiled employment data [13] used to further estimate the potential extent of the ongoing challenges. First, we assessed the number of claims paid, unpaid and deemed non-compensable, by country across southern Africa, by type of claim, gender and year. We then calculated the current rate of claims for each of the southern African countries with at least 25,000 miners who worked in South African mines, taking the 1.6 million miners in The Employment Bureau of Africa (TEBA) database [13] as the denominator. Next, to assess the potential size of unclaimed mining-related lung disease, we calculated expected number of claims by country of origin. To do this we used two approaches: first employing an aggregated rate with a 95% confidence interval established as the weighted mean from various studies that estimated the prevalence of silicosis and tuberculosis in mining populations in southern Africa [28–32], with weights equal to the reciprocal of the study sample variance; and secondly, internally benchmarking against the claim rate from Lesotho, where CCOD outreach activity beyond South Africa had been the most intense. Finally, using the number of expected claims for each country, we further calculated the difference from the actual number of claims to estimate the number of unclaimed cases.

Finally, we apply a critical rights-based lens to examine progress and ongoing barriers, reflecting upon advancements and challenges in terms of institutional accountability as a vehicle for health equity [25].

## Results

Our findings spanned a complex array of interconnected socioeconomic, policy and health issues, from upstream global drivers of the problems identified to various occupational health and safety issues, to specific challenges for individuals in diverse situations. All the interviewees, however, commented on difficulties in ensuring that ex-miners are aware of their rights, and that BMEs are offered, claims are filed, and compensation provided where due.

### Creating a data system

Various sources of information have been tapped to try to track down miners to whom compensation is, or may be, due, and additional ideas were offered as to what other sources could be pursued. One interviewee (Informant #8), for example, suggested searching for mineworkers’ information through the electoral system and cell phone companies. However, as noted by another interviewee (Informant #7):

“*[I]t starts at the source that the problem is identified, which is the medical hub of the mine. There should be some type of a link or something alerting the MBOD that there is a possible claim coming up…So that they can get their sputum results... x-rays… There must be some flag or something because while the person is still with you [at the mine]… they should prepare documentation and get all information they can [in order] to stay in contact with that person. Because the type of disease takes a long time, that’s when your bank account goes dormant, and then the cell phone has expired...We have found, confirmed from the tracing company, that after three months, it [this information] is not effective anymore.”*

This informant then added: “*it’s hard [for the mines] to get information because of the distrust of the people who have historically exploited them*.”

To the extent that data were kept in the past, it had been collected mostly manually and essentially just stored. In 2014 the Chamber of Mines funded an initial project, using TEBA to collate the data of some 200,000 CCOD files, and in 2015, the CCOD launched another project, titled Project Ku-Riha, to retrieve missing information in 100,000 unpaid claims and pay as many as possible. Later another project funded by the gold mining companies collated data from approximately 400,000 MBOD files. In consolidating the files, a new comprehensive CCOD/MBOD electronic database was established – a long overdue accomplishment. As of December 31, 2017, this database contained 111,166 claims that had been paid compensation, (55,864 of which were for permanent lung impairment and another 52,473 for lost earnings due to tuberculosis); it also, however, showed that 107,714 compensable claims remained unpaid, in addition to 141,626 that were deemed non-compensable and the remaining files either deferred due to missing documents or awaiting certification.

**Fig. 1:**
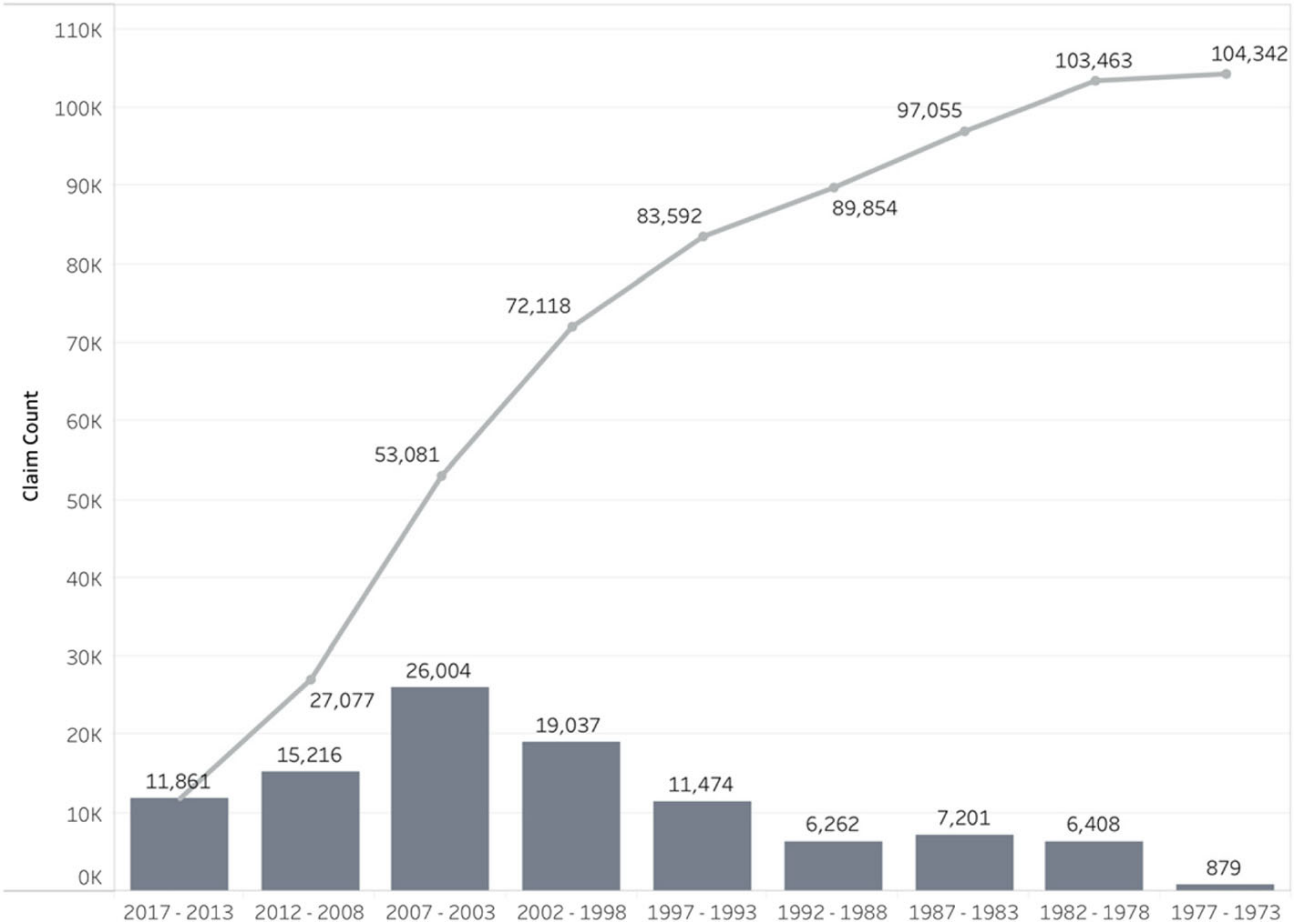
Number of unpaid occupational lung disease claims^a^, showing a running cumulative total^b^, with date of first claim filing increasing to the right, back to ODMWA 1973. ^a^This graph of the 107,714 unpaid claims excludes 3,372 claims due to unknown last benefit medical examination date

Figure 1 shows both the cumulative number of unpaid compensable claims and date of last known BME: 4230 cases dated from before 1980; 10,334 from 1980 to 1989; 25,568 from 1990 to 1999; and 66,964 since 2000. Most of these backlogged claimants (26,717 of the 107,714 unpaid claims) are likely to be deceased, although the precise number is unknown - and there are often dependants who are entitled to compensation.

**Fig. 2:**
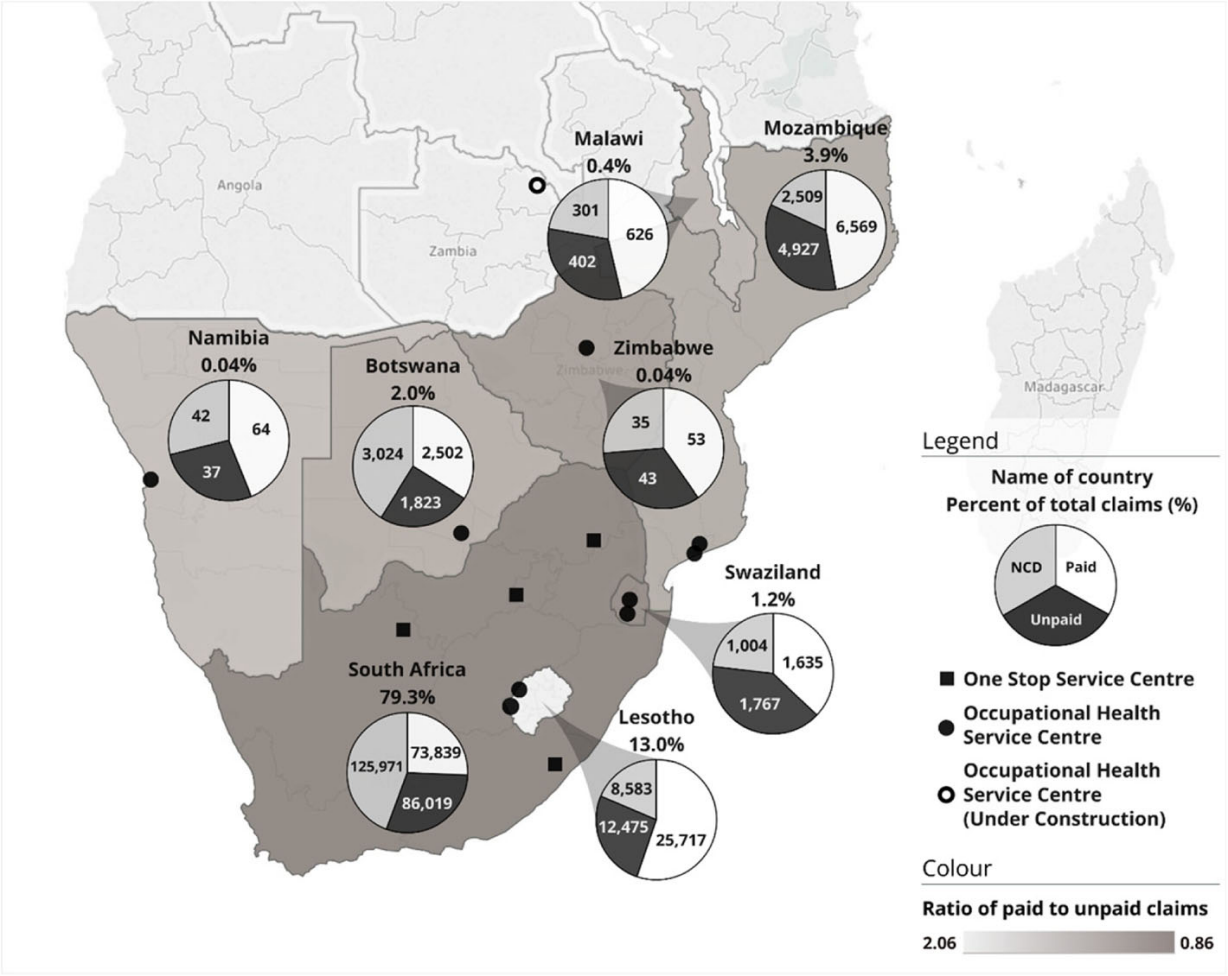
Occupational lung disease claims by worker’s country of origin and claim status, also showing local and mobile service centre locations. ^a^NCD = Non-compensable disease claim. ^b^534 claims (0.1% of the total 360,506 claims) are in countries not shown above. ^c^148,618 claims where the country of origin was not listed were distributed proportionally to claims where the country is known. ^d^An Occupational Health Service Centre, located at Kibong’oto Infections Disease Hospital in Tanzania, lies outside the map above

As shown in Fig. 2, of the 360,506 total claims filed, 281,068 were from ex-miners living in South Africa (i.e. 78.0%), but 7478 were from Botswana (2.1%), 50,593 from Lesotho (14.0%), 1419 from Malawi (0.4%), 4442 from Swaziland (1.2%), with the rest from elsewhere in southern Africa. Of these, 107,714 were unpaid, in which 24.8% (26,707) of the ex-miners were known to have already died. The high ratio of unpaid to paid claims, also shown in Fig. 2, highlights the injustice and militates for intensifying service provision.

### Accessing services and asserting rights

There were differences of opinions expressed amongst our interviewees as to who should be conducting the work to address the backlog of unpaid claims. Informant #1 believed that the tracking and tracing efforts should not only fall to the government:

“*It’s unfortunate that it falls back to government. It’s the mining industry that has created the problem, but it’s government now that has to fix the problem…Government is now going to these communities and telling them they are due benefits. .. [I]t’s the role of the private sector to say we are part of the people that created the issue and loop holes so we are the people to assist*.”

Nonetheless, responding to the legacy of lack of access to BMEs and claims assistance, the CCOD established local “One Stop” service units; two were set up in 2014, one in Carletonville (a current mining area) and the other in Mthatha (a labour sending area). As of December 2017, there were similar “One Stop” centres established in Burgersfort and Kuruman in South Africa and equivalent Occupational Health Units in Botswana, two in Lesotho, two in Mozambique and two in Swaziland – see Fig. 2. As one interviewee noted:

“*…You are tracing people that came to Gauteng to work and might go back to their place of origin – Malawi, Swaziland, Limpopo – How do you track the benefits that are due to them? We don’t have a system where we say we got John from wherever and John is still there.”(*Informant #1*).*

During 2016, mobile services were piloted in eight selected districts in the Eastern Cape. Fifty Community Health Workers from the Eastern Cape Department of Health were deployed to work with traditional leaders and local municipal structures to track and trace ex-miners or their beneficiaries and prepare them for BMEs and claims administration services to be provided by mobile units. During an outreach trip in Maseru, 600 unpaid claimants were identified; the CCOD then organised a mobile unit to go to Lesotho to urge individuals to come for BMEs and complete documentation for compensation.

By the end of 2016, the mobile units had also conducted outreach activities in Botswana, Lesotho, Mozambique, and Swaziland. Through these activities, 2892 BMEs were conducted.

With advocacy and grassroots activism intensifying, high-profile outreach and awareness campaigns for ex-miners were launched, with the Deputy Minister of Mineral Resources, Minister of Health, and Deputy President of South Africa actively involved in efforts to enhance miners’ knowledge of their social protection entitlements (pension, provident, unemployment insurance and compensation funds). Over 80,000 miners or their dependants were thus reached by the end of 2017 in mass meetings in mining and peri-mining communities and labour sending areas, suggesting that some progress was indeed being made.

While substantial progress has been made from the considerable struggle that has ensued, our interviews revealed that there is a persisting lack of awareness in ex-miners of their rights, including their entitlement to BMEs. Even when workers know their rights and come forward, services cannot always be provided. As one Department of Health interviewee (Informant #12) noted:

*“When we went to Matatiele, we had a list of people of 300-400 we wanted to see, we saw 2000 people. We couldn’t identify whether it was the person we are looking for or just a walk-in claim. In Bizana, we had 300 people, and everyone we came to look for were in the database. We managed to see most [but not all] of the people on the three days. There were 188 people on the last day because we could not re-schedule the service.”*

Considerable concern was expressed that if there is no sustained funding, expectations will have been created among the communities that the mobile units will return, which may not be the case, and as the informant noted: “*The same people will continue to come because they are so desperate. We are just flying by the seat of our pants.”*

### Increasing rate of payments

**Fig. 3:**
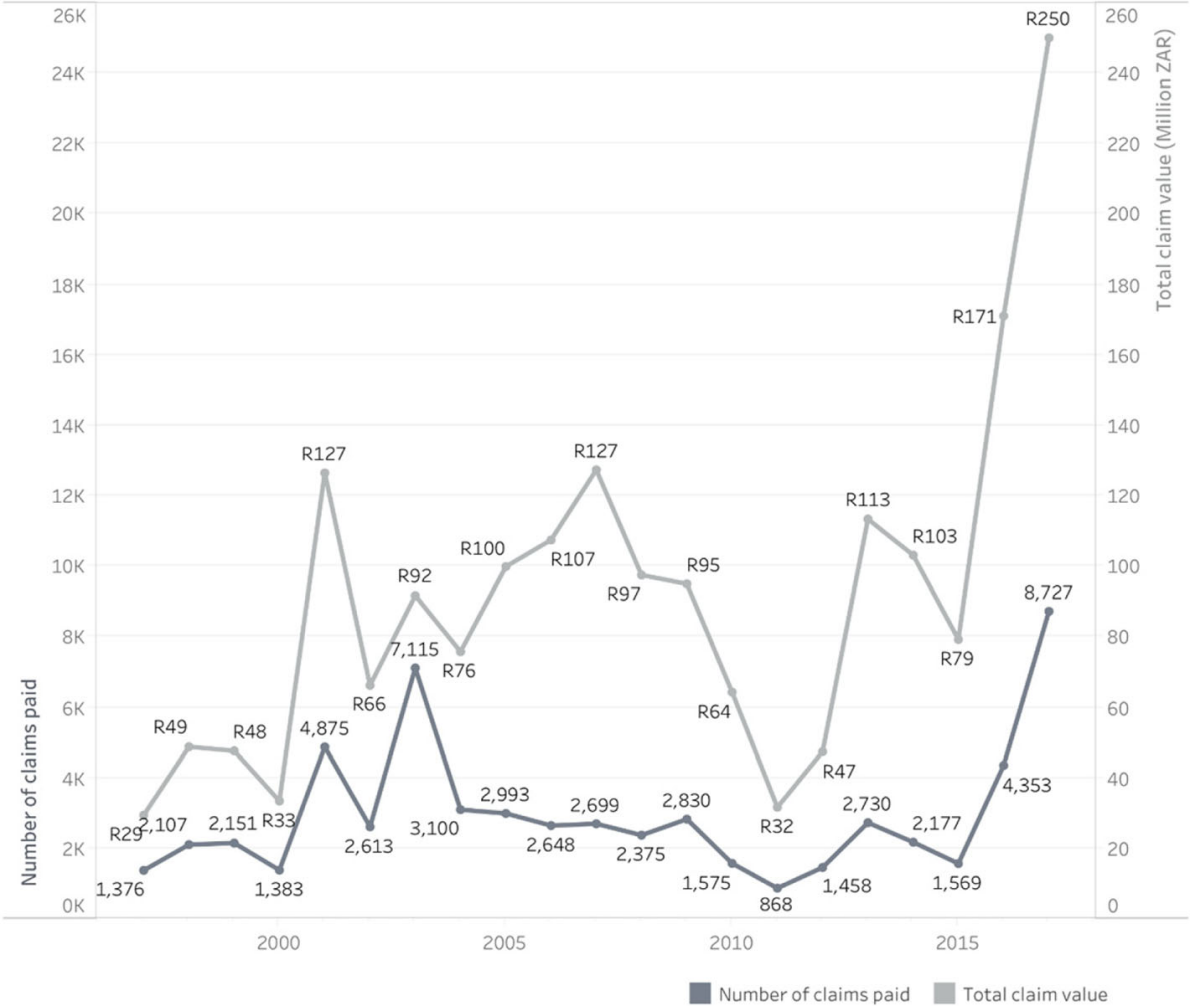
Frequency and value in South African rand (ZAR) of occupational lung disease claims that have been paid. Displayed by calendar year, from 1997 to 2017. Graph includes 61,722 paid claims over 21 years from 1997 to 2017; it excludes 49,444 paid claims over the 24 years from 1973 to 1996

Despite the difficulties and concerns, the number of compensation claims paid has been increasing steadily, as shown in Fig. 3 by year and amount paid. Whereas only 1575 claims had been paid in 2010 and 868 in 2011, claims paid in 2017 numbered 8727. On average, about half the paid claims relate to tuberculosis, and the other half for permanent lung impairment. Undoubtedly the efforts underway are having an important effect.

**Fig. 4:**
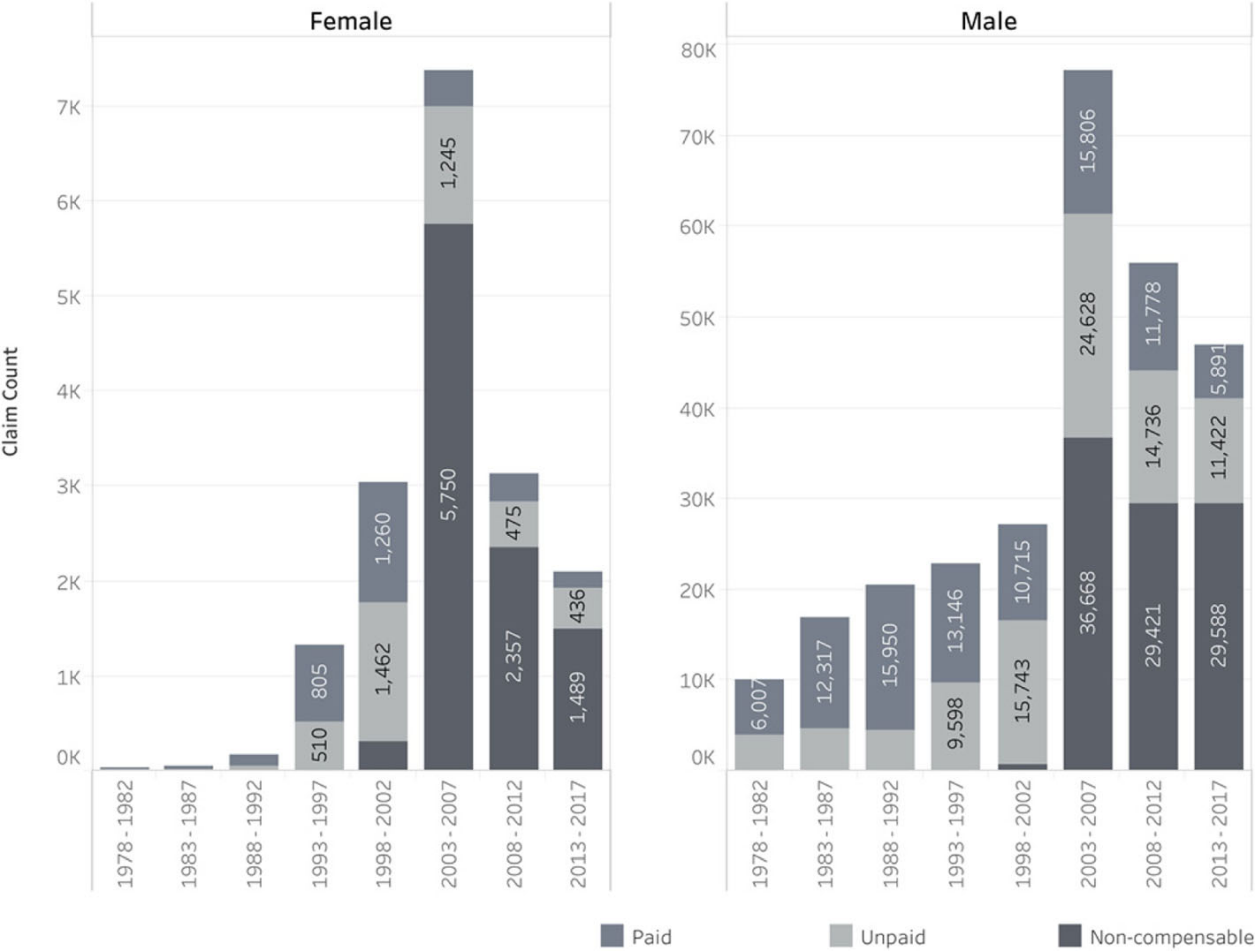
Number of claims, separated by gender, grouped into 5-year periods from 1978 to 2017, shaded by the payment status. ^a^Note the different y-axis scales between females and males; there are approximately ten-fold the number of male compared to female claims. (Records where gender was not recorded were excluded)

Interestingly, the proportion of paid versus unpaid claims among women miners (see Fig. 4) is higher than in men. This higher rate of payment reflects the fact that these claims were more recent, a higher proportion of which were tracked and paid, and that the migrant labour force from countries other than South Africa was almost entirely male, such that the hardest to track group had a much lower proportion of female miners.

The sharp increase in rate of compensation payments to ex-miners clearly demonstrated the feasibility of addressing well-documented injustices once the will to act was pursued and some funding provided for “the big push” for expansion and scale-up in response.^2^ Nevertheless, serious barriers remain to be addressed.

### Providing payment to ex-miners and/or their families for compensable claims

Even with the “One Stop” facilities providing claims services, manual verification of fingerprints is still required for processing payments - a procedure necessitating trained personnel. As one interviewee stated:

*“In Lesotho, you need to pay for fingerprints at the police station. And then of course you have to travel far to go to the police station. There is a cost involved. So that makes it difficult to obtain documentation*.” (Informant #6)

Other challenges relate to name changes in passports, especially from miners from Mozambique and Lesotho. One interviewee noted: “*the same person may have three passports on file*!” Additionally, the CCOD needs a letter of authority from the court to nominate the beneficiaries and open a bank account. Bank accounts usually close after 3 months of inactivity, which is common for out-of-work ex-miners. Although one of the large South African banks agreed to open accounts for 9 months and waive registration fees, the process of obtaining benefits remains difficult, especially when the claimant is deceased and the CCOD needs not only bank account but also identification documents and fingerprints from dependents. One interviewee (Informant #6) described the process encountered by a beneficiary in Lesotho:

“*Before the widow obtains the documents, first of all, they have to deal with family. The family has to witness to say, ‘We know this woman is the wife of the son’. Then the five family members with passports or ID … have to sign the letter and then refer to the area chief to confirm where they are coming from. And the area chief will refer the widow to the district chief. And then from that chief, they will refer the beneficiary to the district administrator. And then the district administrator will refer to the master of courts who will provide the letter. That’s where the second wife will suffer to get the letter because the family will not give the letter. Most beneficiaries will suffer from this because the mineworker is deceased.”*

Even when BMEs are provided, criteria for compensation met, the claim allowed, identification verified, and a bank account opened, calculating compensation due from tuberculosis-related lost earnings can be complicated, requiring computing earnings, including safety and production bonuses. Interviewees reported that mines often get this wrong.

### Promoting the filing of claims

Table 1 outlines various studies conducted to ascertain the prevalence of occupational lung disease in ex-miners. Using our first method to calculate the expected number of claims, and consequently the shortfall (i.e. using aggregated rates from these published studies [28–33]), established a 17.1% ± 1.2% prevalence for silicosis and a 16.8% ± 3.0% prevalence for tuberculosis, for an expected overall compensable claim rate of 33.9% ± 3.3%. In Table 2, we calculated that the rate of compensable claims (both paid and unpaid) based on the CCOD / MBOD and TEBA database, ranged from 3.5% of miners from Malawi to 20.0% of miners from Lesotho. Using our second method (taking 20.0% rate of compensable claims based on the claim rate from Lesotho) relies on the assumption that the rate of claims should not be markedly different between countries, although we are aware that dust exposure may vary considerably amongst the national groups, as miners from different countries were often recruited for particular jobs. However, no exposure measurements are available to inform this assumption. Using these rates and the number of miners from each country in the TEBA database, we find both methods suggest that *more than 100,000 additional miners should have compensable claims*.

**Table 1:** Independent studies of prevalence of silicosis (ILO profusion _≥_ 1/1) and tuberculosis using radiography from a random sample^a^ of ex-miners who worked in South Africa

Study	Population & inclusion criteria	Mean age in years	Mean duration of employment	Size of study^b^	Silicosis % ± 95 CI	TB % ± 95 CI	Both % ± 95 CI
		(range)			(count)	(count)	(count)
Girdler-Brown et al., 2008 [28]	Former gold miners from Lesotho	49.4 (25–61)	25.6	610	24.3% ± 3.4% (148)	30.2% ± 3.6% (184)	10.7% ± 2.5% (65)
Trapido et al., 1998 [30]	Random sample of ex-miners in the Libode district	52.8 (34–78)	9.3	228	20.6% ± 5.3% (47)	32.9% ± 6.1% (75)	–
Steen et al., 1997 [31]	Former miners from Botswana	55.7 (28–93)	13.4	101	25.7% ± 8.6% (26^c^)	26.7% ± 8.7% (27)	–
Hnizdo and Sluis-Cremer, 1993 [32]	Former white gold miners, age 45–54 years, underground service of at least 10 years	52.6 (30–70)	23.5	2235	14.0% ± 1.4% (313)	–	–
Meel, 2002 [29]	Former gold miners from Transkei district	51.6 (35–66+)	12.4	271	33.9% ± 5.7% (92)	61.6% ± 5.8% (167)	28.4% ± 5.4% (77)
Churchyard et al., 2004 [33]	Active black gold miners over 40 years of age in the North West province	46.7 (37–59)	21.8	515	18.3% ± 3.3% (94)	19.6% ± 3.4% (101)	–
Average		51.5	17.7	660	17.1%^d^ ± 1.2%	30.5%^d^ ± 2.1%	13.7%^d^ ± 2.2%

^a^Participant selection within these studies is assumed to be unbiased

^b^Excludes any non-randomly selected participants, or participants for which no data was collected

^c^ILO profusion ≥1/0

^d^Weighted mean is the maximum likelihood estimator of the distribution means

^e^The overall prevalence is *p*_*silicosis*_ + *p*_*TB*_ − *p*_*both*_ = 33.9 %  ± 3.3%

**Table 2:** Number of compensable occupational lung disease related claims for countries in Southern Africa, 1973–2017

Country	Miners^a^ N (% of total)	Compensable claims^b^ N^c^ (% of total)	Rate of claims by country^d^ %	Expected claims: Method 1^d^ N (Difference)	Expected claims: Method 2^e^ N (Difference^f^)
South Africa	1,189,515 (73.3)	159,858 (73.2)	13.4	402,687 (242,829)	237,573 (77,715)
Lesotho	191,225 (18.8)	38,192 (17.5)	20.0	64,735 (26,543)	38,192 (0)
Mozambique	152,091 (9.4)	11,496 (5.3)	7.6	51,487 (39,991)	30,376 (18,880)
Swaziland	31,958 (2.0)	3402 (1.6)	10.6	10,819 (7417)	6383 (2981)
Botswana	29,224 (1.8)	4325 (2.0)	14.8	9893 (5568)	5837 (1512)
Malawi	29,741 (1.8)	1028 (0.5)	3.5	10,068 (9040)	5940 (4912)
Total	1,623,754	218,301	13.4	549,689 (331,388)	324,301 (106,000)

^a^From Ehrlich et al. (2018) [13]. Only includes total number of miners from 1973 to 2013. The estimated present average age of miners in the TEBA database (54 years) matched well with the age of miners in the selected studies (51.5 years); we concluded that the TEBA database would provide adequate denominator data for rough estimation

^b^Includes both paid and unpaid compensable claims

^c^84,596 claims that are missing the country of origin on file have been allocated in proportion with the claims that do have a country listed

^d^Total is 33.9% of the total miners for the country, a target proportion established by aggregate analysis (see Table 1)

^e^Total is 20.0% of the total miners for the country, a target proportion set by Lesotho. Difference subtracts the actual compensable claims made from the total

^f^Difference subtracts the actual compensable claims made from the expected total

### Moving forward with social protection and prevention

The coercive migrant labour system not only strains ties of individuals with their social networks and spreads tuberculosis across borders, but also has been creating disempowering relations and contracts, for example, by designating and providing (often sub-standard) accommodation.

“*If you get recruited for the migrant labour system, you are tied down in the contracts - I can’t stand this working condition - you must see out the contract. It also removes the power to negotiate the worth of one’s value. It’s predetermined by the migrant labour system – ‘we recruit you, we pay you so much, and we give you these types of accommodation and you are restricted to that.’ It’s very disempowering*.” (Informant #5)

Also, choices for health insurance and services have been limited, with the public health system’s insufficient involvement when sick miners are repatriated further aggravating cross-border spread of disease.

*“You’re taking men and putting them in a compound - if you can call it that - or you can call it a hostel – so you’re housing a lot of people in a small locality – so it’s spreading diseases and also taking these diseases and going back to where the mineworkers came back from. Also, if you look at HIV, the other members of the communities are impacted around the mining area”. (Informant #1)*

As noted by the interviewee, *“The public health system is not involved sufficiently in the migrant labour system as it is deemed ‘too political’”*.

Although the Global Fund has been targeting tuberculosis in miners [34], it was clear from our analysis that social development programs, including psychosocial support, which must encompass the needs of dependants such as women and children, are still urgently needed. As one interviewee described the problem of ‘left children’:

“*We went to a home of a man with MDR TB and thirteen children; he was not on treatment, the youngest child was 16 months, and there was malnutrition. Really, there is no support or continuity of care for the family.*” (Informant #12)

Another interviewee (Informant #2) noted:

*“…Maybe the wives now have TB from the miners. Now children have no support for education …– some are sole breadwinners. Some areas have no support for children to get education. Because most of the miners come from remote areas, they need special support of especially children of ex-miners from rural areas.”*

The interviewee emphasized that the problem is not just lung disease and fatalities, but also musculoskeletal injuries, hearing loss, and various serious injuries.

*“Remember if you come back with no legs, your family needs support to deal with that. …Maybe their house does not have support for a wheelchair…. They also need the psychosocial support because they need to accept the situation.”(*Informant #*2)*

Some of our interviewees expressed a view that the unions were not doing enough in the area of health and safety of miners, as noted by this interviewee (Informant #5):*“Our trade unions are very weak on occupational health issues. They are very strong when it comes to wages and things that bring money to the pockets of individuals.”* Several interviewees also noted that the unions do not look after members who are no longer in the workforce, noting that the unions do not track the whereabouts of miners after they leave.

## Discussion

### Towards a rights-based approach: Inclusive and with all hands on deck

Adams et al. [7], in their study of the views of ex-miners, referred to the masking of tuberculosis as extending “beyond knowledge gaps and stigma; it is an articulation of symbolic and structural violence.” Our study extended the documentation of difficulties previously outlined by others [19, 35–37] and applied a rights-based approach requiring distributive as well as procedural justice for all, highlighting the important role of advocates and activists in holding institutions accountable and ensuring that the private sector adequately resources the state to fulfil its role. It was very much in the context of pressure from advocacy groups and activism that the Chamber of Mines, gold mining companies, the World Bank, UKAID and the Global Fund came together to begin to tackle this injustice; yet more is still needed. In this regard we note that while the marginal participation of women in the mining workforce is beginning to be addressed [34] with claims seen from female miners as well, particular attention is also required to actively promote the rights of widows and wives of the almost entirely male migrant workforce to equally assert their rights to compensation and other hitherto elusive social protection funds. Additionally, government cooperation across borders is vital with respect to enforcement of improved labour conditions, medical standards and social protection. While power relations within civil society organizations cannot be ignored [25], the active participation of miners’ unions, along with other advocacy groups is urgently needed, to work alongside government and the mining companies to ensure accountability for the prevention and social protection in the mining sector; the injustices are deep-seated, and progress requires all hands on deck.

### Data system needs

At the heart of a rights-based approach, as noted by London and Schneider [25], is institutional accountability. As the old expression goes “no data, no problem.” To be accountable, responsible institutions must have the data needed to fulfil their mandate. The mining companies must ensure that the state has access to information about its workforce, the work-related medical test results obtained, and the exposure conditions, so that the government can do its job of prevention and social protection. To this end, other sectors, and other countries have well-established human resource databases that can be linked to information on work-related health, exposures incurred, and claims filed; this needs to be done in the South African mining industry as well. With a comprehensive information system platform now being rolled out in the South African health sector [38], prospects are now excellent for establishing systems for providing needed data.

### Seeking a fair and sustainable revenue stream

Understanding the current developments and ongoing challenges is especially important as gold mining companies are currently confirming terms of a soon-to-be-settled class action on silicosis and tuberculosis^3^; identifying eligible miners and ex-miners across the region will be an important part of the class action settlement process in the gold mining industry [39].

As has been noted in previous analyses [8, 10], a severe limitation to providing compensation for ex-miners has been chronic underfunding of service delivery systems – and the industry’s argument that their profit margins did not allow them to cover full costs was judged to be specious by a court judgment in 2010 [10]. Indeed, growing concerns over accounting practices such as “transfer pricing” - whereby extractive industries avoid paying appropriate levies by understating value creation in settings where mining occurs while arbitrarily assigning increased “value” to transactions in tax haven settings - have led to calls to end this and broader practices of Illicit Financial Flows (IFFs) [40]. Citing an Oxfam report entitled “*Rigged Rules and Double Standards: Trade, Globalization and the Fight Against Poverty*” [41], Ooms and colleagues, in a previous issue of this journal, argued that classic free trade theory never considered the current within-firm trading systems and oligopoly/monopoly or trade rule-bending mechanisms exhibited in the modern economic and political systems [42]. Such mechanisms used by multinational corporations, also known as “profit shifting” has been well-documented to have devastated health systems elsewhere in Africa as well [43]. Work currently underway to characterize the extent of IFFs in South Africa and promote practices that ensure sufficient “domestic resource mobilization” [44] is central to understanding how financial resources needed to promote social justice can be sustainably provided. The impact of transfer pricing alone has been estimated to be in the order of a loss of $500 million to $2 billion USD from the South African economy [45]. According to a recent Global Financial Integrity report, South Africa lost over $100 billion dollars from various IFFs over the period 2002–2011 [46]. In light of evidence of purposeful underfunding of national systems by global corporations pursuing tax avoidance strategies, the argument that South Africa is too poor to provide the social protection needed is simply not tenable. The cross-border impact of the underfunding – which has been undermining health equity and social justice not only within South Africa but across the entire southern African Region - needs particular scrutiny.

Ooms and colleagues [42] argued that a “big push” to address the chronic underfunding of health systems in Africa must be replaced by a more sustained approach. In a similar vein, in presenting data on the difficulties faced by ex-miners including quantification of unpaid and estimated unfiled claims by miners in the context of the globalized mining sector, we argue that it is in keeping with human rights obligations to provide social protection, including compensation to miners across southern Africa who developed occupational lung disease, and, importantly, to prevent further such cases. Furthermore, we argue that the underlying financial inequities must be addressed and the barriers to accessing compensation and other social protection rights need to be contextualized in larger concerns about capital flight, worker rights, labour relations and corporate practices and policymaking.

## Conclusion

While considerable challenges remain in providing compensation and prevention services to all workers and their families eligible to receive these, the achievements we report here in providing compensation for occupational lung diseases suffered by miners in South Africa provides testimony to what funded systems can contribute to addressing injustices, when held accountable to do so. Over 15 years ago it was observed that: “The social and environmental contexts that determine disease are no longer simply domestic but increasingly global. Greater attention in research is required to the linkages between these issues and to their economic and political drivers that are, like the issues, increasingly global in scope” [47]. Indeed “taxation and tax havens” were identified well over 15 years ago [47, 48] as contributing profoundly to underfunding health systems such as those needed for actions to address occupational lung disease among ex-miners in South African mines, thereby compounding the historical colonial roots of this injustice. Adoption of measures by mining companies and national governments to block such evasion of responsibility is thus a necessary step in enabling the sustainable pursuit of remediation.

Disease-prevention measures are also needed to complement the strengthening of systems to facilitate access to adequate health, social and financial services, including receipt of compensation. Such are manifestations of the macro-level structural processes that need to be considered when assessing extractive sector impacts. As Bambas-Nolen et al. [48] argue, an effective response to the health impacts of mining must encompass *“*meaningful solutions to the myriad impacts of social determinants of health shaped by extractive industries.” The overall balance sheet of burdens and benefits of mining must be examined over the life cycle, with serious commitment to improving working and living conditions as well as finding and providing compensation to those to whom it is due. Such measures must be reflected in all relevant international and national policy agendas (e.g. Mining Charters) – and actively pursued in a transparent manner that does not perpetuate and entrench asymmetries of power between governments, mining companies, and the affected miners and their families.

In the context of lawsuits and grassroots struggles, recent developments indicate that progress is possible. The cash transfers won to date can help alleviate poverty and restore the dignity of miners and their families; however, obstacles need to be overcome. Moreover, prevention interventions such as dust control are important to ensure that future generations of workers have “decent work” and do not develop occupational lung diseases. While recent developments in providing compensation can be considered an important start, much more is needed, particularly to support the many migrant miners, ex-miners and dependents in southern Africa who may not know their rights or who are encountering complicated barriers to compensation and justice. London and Schneider [25] argue that globalisation, while shaping injustices, also opens possibilities to use international human rights benchmarks to harmonise regulatory standards upwards, improve health systems, and restructure trade and taxation, noting that nongovernmental community-based organizations, as well as the media and “networks that cut across class and social location” are required to ensure political accountability. In this regard, promoting the tackling of occupational lung disease from the mining sector as a human rights issue requires that all segments of society hold the government accountable, and that an adequate sustainable revenue source from the mining sector is allocated to support state efforts to act.

## Footnotes

1 The history of Dr. Randeree’s association with both South African (BK), serving as Director General of Health in the Northern Cape at that time, and international (AY and JS) members of our team, with whom he was working in Canada, is illustrative of how international solidarity dimensions of resistance to global forces driving health inequities can be operationalized

2 As this revised article is being submitted, we note that there were 1203 payments for the month of March 2018 amounting to R18.2 m - the highest number of payments in a month since the commencement of tracking and tracing.

3 As we submit this revised article, we note that the affected gold mining companies and the claimant lawyers have reached an out-of-court settlement amounting to approximately $400 million USD. The settlement covers miners since 1965 and dependents with compensation for claims for TB and silicosis. Additional funds amounting to approximately $70 million USD has been set aside for the administration, tracking and tracing of claimants or dependents and provision of BMEs over the next 12 years. https://www.reuters.com/article/us-safrica-mining-silicosis/south-africa-miners-reach-400-million-silicosis-settlement-with-mining-companies-idUSKBN1I41B9.
