# Correction to: Tackling injustices of occupational lung disease acquired in South African mines: recent developments and ongoing challenges

**DOI:** 10.1186/s12992-018-0399-9

**Published:** 2018-08-01

**Authors:** Barry Kistnasamy, Annalee Yassi, Jessica Yu, Samuel Spiegel, Andre Fourie, Stephen Barker, Jerry M. Spiegel

**Affiliations:** 1 Department of Health, Johannesburg, South Africa; 2 School of Population and Public Health (SPPH), University of British Columbia (UBC), 430-2206 East Mall, Vancouver, BC V6T 1Z3 Canada; 3 Centre of African Studies, University of Edinburgh, Edinburgh, UK

## Correction

Following publication of the original article [1], the author flagged that unfortunately in endnote no. 1 it read "serving as Director General of Health", when it should be "serving as Deputy Director General of Health". As such, endnote no. 1 of the original article [1] has now been corrected to reflect this.
